# Current progress of immune checkpoint inhibitors in the treatment of advanced hepatocellular carcinoma

**DOI:** 10.1042/BSR20212304

**Published:** 2022-02-07

**Authors:** Xiaoqiang Yin, Tongchui Wu, Yadong Lan, Wulin Yang

**Affiliations:** 1 Department of Hepatobiliary Surgery, Hefei Cancer Hospital, Chinese Academy of Sciences, Hefei, 230031, Anhui, China; 2 Anhui Province Key Laboratory of Medical Physics and Technology, Institute of Health and Medical Technology, Hefei Institutes of Physical Science, Chinese Academy of Sciences, 350 Shushanhu Road, Hefei, 230031, Anhui, China; 3 Medical Pathology Centre, Hefei Cancer Hospital, Chinese Academy of Sciences, Hefei, 230031, Anhui, China

**Keywords:** biomarkers, hepatocellular carcinoma, immune checkpoint inhibitor, immune response, immunotherapy

## Abstract

Hepatocellular carcinoma (HCC) is the most common primary liver cancer worldwide. The onset of the disease is occult and develops rapidly. As a result, the disease is often detected when it is already in advanced stages, resulting in patients losing the best opportunity for liver transplantation and surgical treatment. Therefore, effective treatment of HCC is particularly important in clinical practice. During the past decades, there have been considerable advances in the treatment of HCC, and immunotherapy is increasingly recognized as a promising approach in clinical trials. In this review, an overview of immune checkpoint (ICP) inhibitors (ICIs) and their role in the treatment of liver cancers, particularly advanced HCC, is presented and the recent therapeutic progress with treatment with different ICIs alone or in combination with other methods/therapeutic agents is summarized. In addition, the identification of biomarkers to predict treatment response and the limitations of current ICIs are analyzed, and future directions for ICI treatment are discussed.

## Background

Liver cancer is the sixth most common type of malignant tumor and the third leading cause of cancer-associated deaths [1,2]. Hepatocellular carcinoma (HCC) accounts for approximately 75–85% of primary liver cancers and is currently one of the most common malignant tumors, which seriously threatens people’s lives and health. The vast majority of HCC cases occur in the setting of chronic liver disease, with cirrhosis being the primary risk factor for HCC. Currently, surgical treatment is still the main approach to obtain a radical cure for HCC, including early hepatectomy and liver transplantation, with a 5-year survival rate of up to 70% [3]. For patients with advanced HCC, various non-surgical treatments such as transcatheter arterial chemoembolization (TACE), local ablation therapy, radiotherapy, and molecular targeted drug therapy with sorafenib can be selected [4–8]. However, the treatment of advanced HCC is still insufficient and the tumor is prone to invasion, metastasis, and recurrence, resulting in a low overall survival (OS) rate, high mortality, and a poor prognosis.

In recent years, with the rapid development of molecular immunology, tumor immunotherapy has come into being, providing a new option for the treatment of HCC. Tumor immunotherapy refers to the use of the immune defense mechanism of the body to enhance the antitumor immune response and overcome the immune escape of the tumor through various methods, thereby controlling and killing tumor cells. The current progress of tumor immunotherapy manifests itself primarily in the immune checkpoint (ICP) inhibitor (ICI), and is mainly represented by tumor vaccine therapy (dendritic cell [DC] vaccine and oncolytic virus vaccine), and adoptive cell therapy (ACT); among them, ICI therapy is of particular concern and has achieved positive results [9,10]. In September 2017, the U.S. Food and Drug Administration (FDA) approved nivolumab for patients with HCC previously treated with sorafenib, marking the official arrival of the era of immunotherapy for liver cancer.

The mechanisms of tumor immune escape have been intensively explored [11–13]. Tumors can induce and establish a tumor microenvironment (TME) conducive to immunosuppression, including immunosuppressive cells and molecules, resulting in the loss of antitumor function of T cells and triggering immune escape. Regulatory T cells (Tregs) and myeloid-derived suppressor cells (MDSCs) are the most important components of immunosuppressive cells. Furthermore, immunosuppressive molecules include ICPs, such as the programmed cell death 1 (PD-1) receptor and its ligands programmed death ligand 1 (PD-L1) and PD-L2, as well as the cytotoxic T lymphocyte-associated antigen 4 (CTLA-4) [14]. ICIs, including anti-PD-1, anti-PD-L1, and anti-CTLA-4 antibodies, can enhance the activities of effective T cells and inhibit immunosuppression in the TME [15,16]. Furthermore, lymphocyte activation gene-3 (*LAG-3*), as an ICP control protein, has its main function in negatively regulating T-cell immunity. It is expected to become the primary target, second only to PD-1 in the development of cancer treatment [17]. T-cell immunoglobulin mucin-3 (TIM-3) was first identified as an immunosuppressive molecule on the surface of T helper 1 (Th1) cells [18], and animal studies involving gene knockout and tumor-bearing mouse models have shown that compared with treatment with anti-CTLA-4 and anti-PD-1 antibodies, the anti-TIM-3 antibody (Ab) does not cause obvious autoimmune side effects, suggesting that it has good prospects for clinical application [19]. The T-cell immunoglobulin and ITIM domain protein (TIGIT) is also a type I transmembrane protein that is expressed mainly in activated T cells, Tregs, memory T cells, and NK cells. TIGIT is usually co-expressed with LAG-3, TIM-3, and PD-1. They jointly participate in the immune recognition of the body and are closely related to patient survival [20]. In summary, ICP offers new hope to patients with advanced HCC [21]. Here, we review the latest research on the mechanism and clinical application of ICIs in the occurrence and development of HCC. New biomarkers for predicting treatment response are described, and the future direction of ICP therapy has been prospected.

## Immune escape mechanisms for immunotherapy in HCC

**Figure 1:**
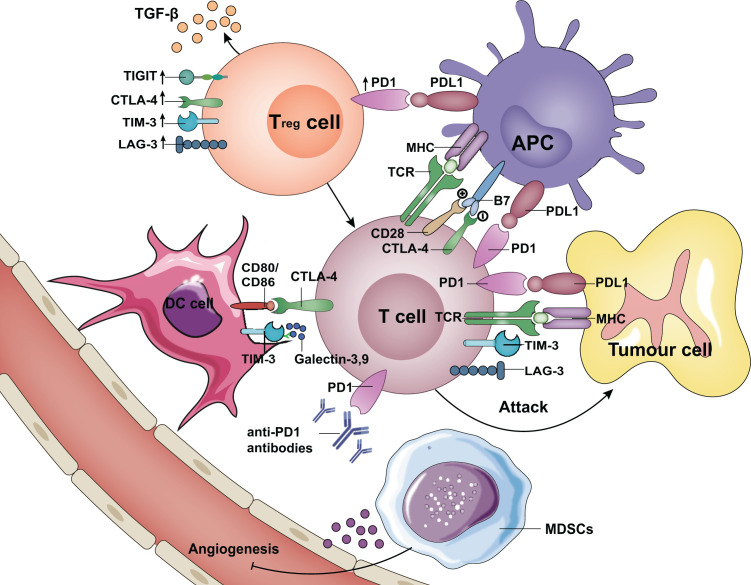
Schematic diagram of immunosuppressive cells (Treg and MDSCs), ICP ligands (PD-L1, CD80/CD86, B7, and galectin-3) interacting with their cognate receptors (PD-1, CTLA-4, TIM-3, LAG-3) to inhibit T-cell activation

The blood supply to the liver comes from portal veins and hepatic arteries. The blood in the portal veins and hepatic arteries contains autoantigens and endogenous antigens, respectively. When a variety of autoantigens and endogenous antigens flow through the liver, autoimmune tolerance is established that prevents the liver from being damaged by the autoimmune reaction [22]. Due to this immune tolerance mechanism, tumor cells in the liver can more easily escape the immunity of the body, avoiding being recognized and killed under the surveillance of the immune system. Furthermore, in the TME, various immunosuppressive cells or molecules form a complex regulatory network to promote tumor cell immune tolerance and escape the body’s immune surveillance (Figure 1). Immunosuppressive cells, such as the increase in Tregs in peripheral blood and tumor-infiltrating lymphocytes (TILs) in patients with liver cancer, can promote transforming growth factor-β (TGF-β) or increase the inhibitory regulatory molecules CTLA4 and PD-L1, further inhibiting the immune response [23,24]. In cancer, myeloid cell differentiation often changes, producing a group of MDSCs to promote angiogenesis and immunosuppression in the TME [25]. Some clinical studies indicate that targeting MDSCs can significantly improve the antitumor effects of sorafenib and ICIs [26]. ICPs, as an important class of immunosuppressive molecules, are expressed on the membrane of different types of immune cells, such as natural killer cells, DCs, tumor-associated macrophages, monocytes, and B and T cells [27]. These ICP proteins function as physiological inhibitors to prevent the activation of these cells, avoiding possible off-target tissue damage. Studies have found that the high expression of ICPs in HCC is related to tumor invasion, progression, and a poorer prognosis. After suppressing ICP expression in HCC models, the *in vivo* growth of HCC is largely inhibited [28,29], and the high expression of ICPs appears to be the key point for HCC to produce immune tolerance. The ICP CTLA-4 has a high degree of homology to CD28, so it can compete with CD28 for the binding site of the B7 molecule on the antigen-presenting cell (APC) surface, thus exerting the function of inhibiting T cells. In addition, CTLA-4 is also expressed on the Treg cell membrane. It inhibits T-cell activation by enhancing Treg activity and differentiation. Treg s derived from liver cancer can interfere with DC function and down-regulate CD80/CD8 expression on DCs in a cell contact-dependent manner *in vitro* [30]. Programmed death receptor-1 (PD-1) is a type I transmembrane glycoprotein located on the surface of T cells. It is also expressed on activated B cells and myeloid cells. PD-L1/PD-L2 are PD-1 ligands expressed on a variety of cells. When PD-L1/PD-L2 binds to its receptor, its downstream tyrosine residues are phosphorylated, thus recruiting protein tyrosine phosphates (PTPs), such as SHP2, to dephosphorylate key kinases in downstream pathways, such as ZAP70, P13K-AKT, and RAS-ERK. Currently, several ICI clinical trials conducted in the field of advanced HCC have shown that the objective response rate (ORR) of PD-1/PD-L1 inhibitor monotherapy can reach 10–20%, and is safe and reliable [31]. It was recommended in the treatment guidelines as a second-line treatment for advanced HCC.

## PD-1/PD-L1 inhibitors

HCC usually occurs in the context of liver inflammation, in which PD-1 is highly expressed in intrahepatic lymphocytes, while its ligands, PD-L1 and PD-L2, are highly expressed in Kupffer cells, sinusoidal endothelial cells, and leukocytes once cells are exposed to pro-inflammatory cytokines [32]. PD-1 inhibitors interfere with the binding of PD-1 to PD-L1 and PD-L2 and promote the recognition and killing of tumor cells by the immune system. PD-1 inhibitors have been demonstrated to be effective therapeutics in melanoma, non-small-cell lung cancer (NSCLC), renal cell cancer, bladder cancer, and head and neck squamous cell carcinoma [33]. Nivolumab is the first fully humanized IgG4 monoclonal antibody against PD-1. In 2017, the U.S. FDA approved it for use in patients with advanced HCC who relapsed or became intolerant after sorafenib treatment. In the phase I/II CheckMate040 dose escalation and expansion trial, nivolumab was investigated in 262 patients with unresectable liver cancer (Table 1). The patients included those who were initially treated with sorafenib. The results indicated that the ORR in the dose-expansion group was 20% and the median progression-free survival (PFS) was 4.0 (2.9–5.4) months. In the dose-escalation group, the median OS was 15.6 (13.2–18.9) months. Treatment-related adverse events (AEs) of grade 3–4, including fatigue and diarrhea, occurred in 18 and 23% of the patients in these two groups, respectively, suggesting that the safety of nivolumab is manageable and no new symptoms were observed in patients with advanced HCC [34]. Pembrolizumab is another PD-1 monoclonal antibody. In the KEYNOTE-224 study, 104 patients with advanced HCC who were intolerant to sorafenib or had imaging progression after treatment received pembrolizumab, with an ORR of 17% and a disease control rate (DCR) of 62%, and an mPFS and mOS of 4.9 and 12.9 months, respectively. At the data cut-off date, 17 patients (16%) were still receiving pembrolizumab treatment. Common grade 3 treatment-related events include elevated transaminases and fatigue. Immune hepatitis occurred in 3% of the patients, but there was no viral outbreak [35]. These results indicate that pembrolizumab is effective and tolerable in patients with advanced HCC who had previously been treated with sorafenib. Although pembrolizumab did not reach the established statistical difference, the prolonged trend in OS and PFS suggests that patients can benefit from treatment [36]. Atezolizumab is a new monoclonal antibody that targets the PD-L1 protein. Atezolizumab binds to PD-L1 expressed on tumor cells and tumor-infiltrating immune cells, blocking its interaction with PD-1 and B7.1 receptors. By inhibiting PD-L1, T cells can be activated to destroy tumor cells. In the IMbrave150 experiment, atezolizumab-bevacizumab achieved good results in patients with advanced liver cancer [37]. In addition, camrelizumab, durvalumab, avelumab, and other ICIs have been reported in various experiments, and more first-line treatment options may be developed in the future (Table 1) [34–46].

**Table 1:** Outcomes of clinical trials of ICIs in HCC

Drug	Trial name	Phase	*n*	OS, months	PFS, months	ORR, %	DCR, %	irAER, %	References
**Anti-PD-1**									
Nivolumab	CheckMate040	I/II	214	15.1	4	20	64	25	[34]
Nivolumab /sorafenib	CheckMate459	III	371/372	16.4/14.7	3.7/3.8	15/7	55/58	22/49	[38]
Nivolumab plus Ipilimumab	CheckMate 040	I/II	148	22.8	12.5/22.8	27/32			[43]
Pembrolizumab	Keynote224	II	104	12.9	4.9	17	62	15	[35]
Pembrolizumab	NCT02658019	II	29	13	4.5	32	46		[44]
Pembrolizumab/placebo	Keynote240	III	278/135	13.9/10.6	3.0/2.8	16.9/4.4	62.2/53.3	18.6/7.5	[36]
Camrelizumab	NCT02989922	II	217	13.8	2.1	14.7	44.2	22	[39]
**Anti-PD-L1**									
Durvalumab	NCT01693562	I/II-	39	13.2	2.7	10.3	33	20	[40]
Durvalumab	NCT02519348	I/II	104	13.6	2.07	10.6		20.8	[45]
Atezolizumab plus bevacizumab	NCT03434379 (IMbrave150)	III	336	67.2	6.8			56.5	[37]
Atezolizumab plus Bevacizumab	NCT02715531	Ib	104	17.1	12.4				[46]
Avelumab	NCT02395172	III	396	11.4				10	[41]
**Anti-CTLA-4**									
Tremelimumab	NCT01008358	II	20	8.2	6.5	17.6	76.4	45	[42]

Abbreviations: irAER, incidence of grade 3 immune-related adverse event; ORR, objective response rate.

## CTLA-4 inhibitors

CTLA-4, the first clinically targeted ICP receptor, is a T lymphocyte surface protein that regulates the amplitude of T-cell activation at the initial stage [47]. CTLA-4 inhibitors (such as ipilimumab and tremelimumab) improve the activity of APC and T lymphocytes to recognize and eliminate tumor cells. An NCT study indicated that after treatment of 20 patients with advanced HCC with tremelimumab, the ORR was 17.6% and the DCR 17.6%, with a median PFS of 6.48 months. A significant drop in viral load was observed when new emerging variants of hypervariable region 1 of hepatitis C virus (HCV) replaced the predominant variants present before therapy. It was assumed that this antiviral effect is associated with an enhanced specific anti-HCV immune response [42]. A multicenter randomized phase III study (NCT04039607) investigating nivolumab in combination with ipilimumab as the first-line treatment was scheduled to be completed in September 2023. The FDA has approved nivolumab plus ipilimumab for the treatment of advanced HCC in patients who were previously treated with sorafenib in 2020 [48]. However, this class of immunotherapy has not been approved as a single-agent therapy in HCC.

## Combination therapy of ICIs

PD-1/PD-L1 and CTLA-4 inhibitors can be used alone or in combination with other ICIs to treat malignant tumors. For certain types of solid tumors, combination therapy has a favorable therapeutic effect. For example, when durvalumab (anti-PD-L1 antibody) plus tremelimumab (anti-CTLA-4 antibody) was used to treat primary HCC, ORR increased by 47% compared with durvalumab alone [49]. Since these two antibodies act at different checkpoints, they may have therapeutic synergies. In the CheckMate040 subcohort study, 148 patients who received sorafenib were treated with nivolumab plus ipilimumab. The results suggested that ORR was 31% with increased OS compared with sorafenib alone. Furthermore, the combination of nivolumab plus ipilimumab was determined to have manageable safety, a promising ORR, and a durable response [43]. PD-1 inhibitors alone may not be able to activate a sufficient number of T lymphocytes, and combined use with CTLA-4 inhibitors may further increase the number of activated T lymphocytes, leading to more T lymphocytes in the TME and increased antitumor activity. Therefore, various combination therapies should be further explored to improve therapeutic efficacy.

## ICIs combined with molecularly targeted drugs

In recent years, the combination of a variety of molecularly targeted drugs with different mechanisms of action to control the progression of advanced tumors has become a hot topic for researchers. In 2007, sorafenib, a tyrosine kinase inhibitor (TKI), was demonstrated to improve OS in the Sorafenib HCC Assessment Randomized Protocol and Asia-Pacific trials, opening the door for its use in HCC treatment [4]. It has dual antitumor effects. It not only directly inhibits tumor cell proliferation by blocking the RAF/MEK/ERK-mediated signal transduction pathway but also indirectly inhibits tumor cell growth by blocking VEGFR and platelet-derived growth factor (PDGF) receptors, thereby cutting off tumor blood vessel formation. Clinical trials of anti-PD-1 antibodies combined with TKI, including sorafenib (NCT03211416, NCT03439891, NCT02988440), regorafenib (NCT03347292), cabozantinib (NCT03299946 and NCT01658878), and axitinib (NCT03289533), have shown that, compared with monotherapy with TKI, combination therapy could provide more survival benefits in patients with advanced HCC. Bevacizumab is a monoclonal antibody that targets vascular endothelial growth factor (VEGF) and inhibits angiogenesis and tumor growth. The latest data from the phase III clinical study IMbrave150 [37] were announced at the 2019 ESMO-ASIA. OS at 12 months was 67.2% with atezolizumab-bevacizumab and 54.6% with sorafenib. The median PFS was 6.8 and 4.3 months in the respective groups. Grade 3 or 4 AEs occurred in 56.5% of 329 HCC patients who received at least one dose of atezolizumab-bevacizumab and in 55.1% of 156 patients who received at least one dose of sorafenib.

## ICIs combined with local regional therapy

Local regional therapy (LRT) of HCC is an important method for the systemic treatment of advanced liver cancer, including TACE, radiotherapy, and radiofrequency ablation (RFA). Generally, tumors with low mutation loads are less immunogenic and insensitive to ICI treatment. LRT creates conditions for the release of tumor-associated antigens by directly destroying tumor tissues, thus activating tumor antigen phagocytosis mediated by APCs, activating T lymphocytes, and improving sensitivity to ICIs [27].

### ICIs combined with radiotherapy

Radiotherapy is an important treatment for advanced HCC. It promotes immunogenic cell death and releases antigens from irradiated tumor cells, subsequently activating the immune system. At the same time, radiation changes the TME to some extent and alters the immune resistance of tumor cells. Chiang et al*.* [50] reported that stereotactic body radiotherapy combined with nivolumab for the treatment of unresectable HCC achieved an ORR of 100%. Among the five treated patients, two achieved a complete response and the remaining three achieved a partial response. The median rate for the reduced diameter was 38.7% (30.5–84.5%), the median PFS was 14.9 months, and the 1-year OS and the 1-year local control rate were 100%. A patient had grade 3 toxicities (pneumonitis and skin reaction). There was no classical radiation-induced liver disease. Yu et al*.* [51] applied radiotherapy during nivolumab treatment in 76 patients and found that patients receiving combined therapy had significantly higher PFS and OS than those receiving nivolumab alone. The results indicated that the combination of chemotherapy with ICIs and radiation therapy is effective for advanced HCC and should be further investigated.

### ICIs plus TACE

The liver has a dual blood supply from the portal vein and the hepatic artery, and liver cancer is frequently hypervascular. Approximately 90% of the blood supply of the tumor is supplied by the hepatic artery, a blood supply that is much greater in livers with cancer than in normal livers. Based on this characteristic, TACE, a technique that combines intraarterial chemotherapy and selective ischemia, has been used as first-line treatment for patients with intermediate-stage HCC, including those with large or multinodular HCC [5]. TACE is also used to treat patients with unresectable HCC, although long-term survival remains low [52]. Current ongoing trials include the evaluation of the combination of pembrolizumab with TACE for the treatment of advanced HCC (NCT03397654) and nivolumab combined with drug-eluting beads (DEBs)-TACE (NCT03143270) [53], and the results are expected to be announced in the near future.

### ICI combined with RFA therapy

RFA therapy is currently one of the main interventional treatments for liver cancer. Previous studies have shown that radiofrequency thermal ablation stimulates NK cells, giving them more differentiated and activated phenotypic characteristics, and generally increases their functional activity, improving the antitumor immune response [54]. Duffy et al*.* [55] reported that tremelimumab in combination with tumor ablation in patients with advanced HCC is feasible and leads to the accumulation of intratumoral CD8+ T cells. Patients were enrolled in this pilot study of tremelimumab at two dose levels (3.5 and 10 mg/kg) and were treated every 4 weeks for a total of six doses, followed by 3 months of infusions until the off-treatment criteria were met. Trimelimumab combined with RFA resulted in partial remission in 26% of HCC patients, resulting in probabilities of PFS at 6 and 12 months of 57.1 and 33.1%, respectively, with a median time to tumor progression (TTP) of 7.4 months (95% CI: 4.7–19.4 months). The median OS was 12.3 months (95% CI: 9.3–15.4 months).

## Real-world studies of ICIs

While randomized controlled trials (RCTs) are commonly used to evaluate the safety and efficacy of new drugs, the inclusion and exclusion criteria of RCTs are often too restrictive and the results may not fully conform to the real clinical environment. Real-world research, which aims to generate reliable data on patient responses to drugs in real diagnostic and therapeutic settings, maybe a more appropriate source of data on the safety and efficacy of new drugs. For ICIs, many real-world studies have been performed where large cohorts were analyzed (Table 2) [56–61]. Scheiner et al*.* [57] conducted an international multicenter real-world cohort study with 65 patients of Child-Pugh class A/B/C (34 nivolumab and 31 pembrolizumab). The results indicated that both inhibitors have promising efficacy and safety in patients with advanced HCC, including subjects with Child-Pugh stage B and patients with intensive pretreatment. Chen et al*.* [58] found that patients treated with lenvatinib plus ICI had a significantly higher ORR (41.5 vs 20.0%, *P*=0.023) and DCR (72.3 vs 46.7%, *P*=0.009) than those treated with lenvatinib. A real-world study by Sung et al*.* [59] showed that nivolumab treatment seems clinically effective in treating unresectable HCC in an endemic area of HBV infection. Twenty-nine patients (88%) in this cohort were HBsAg positive. These patients were evaluated for efficacy and showed an ORR of 21.4%. The median OS was 26.4 weeks. Liụ et al*.* [60] also found that the combination of anti-PD-1 immunotherapy plus TKIs proved to be a safe and effective treatment for advanced HCC. Xie et al*.* [61] included 60 patients treated with sintilimab plus TKI between February 2019 and December 2019, showing an ORR of 36.7% (95% CI: 24.9–48.5%) and a DCR of 81.7% (95% CI: 71.9–91.5%). A total of 46 patients (76.7%) reported AEs, and 8 patients (13.3%) discontinued combination therapy due to grade 3/4 serious AEs.

**Table 2:** Outcomes of real-world studies of ICIs in HCC

Drug	*n*	OS, months	PFS, months	ORR, %	DCR, %	References
Anti-PD-1 agent	55	15	10	22	89	[56]
Nivolumab/pembrolizumab	34/31	11.0	4.6	12/49		[57]
Lenvatinib + PD-1 inhibitors	65	14	8.0	41.5	72.3	[58]
Nivolumab	33	6.2				[59]
HAIC + anti-PD-1 antibodies + TKIs	27		10.6	63.0	92.6	[60]
Sintilimab + TKI	60		12.8	36.7	81.7	[61]

Abbreviations: DCR, disease control rate; HAIC, hepatic artery infusion chemotherapy; ORR, objective response rate.

## Biomarkers to predict ICP response

Studies have indicated that the ORR of ICIs is ∼20% in HCC, which means that a considerable proportion of patients do not respond to this type of treatment. Therefore, appropriate patients should be selected for ICI treatment to achieve a higher ORR. If the population likely to benefit can be screened using predictive biomarkers, the advantages of ICI treatment can be better exploited. The tumor mutation burden (TMB) is an index of the total number of mutations in each coding region of the tumor genome. Theoretically, tumors with higher TMB levels can express more neoantigens, thus eliciting a stronger antitumor immune response and may be targeted for immunotherapy. A high TMB and neoantigen load can predict the response of tumors such as melanoma and NSCLC to anti-PD-1 treatment [62]. Unfortunately, this is not the case in HCC. TMB testing on 755 patients with HCC, of which 74% of patients had TMB of <4 mutations/Mb, and 95% of patients had TMB <10 mutations/Mb, suggested that TMB in HCC was at a low level [63]. Microsatellite instability (MSI) is a hypermutation phenotype caused by mismatch repair defects (dMMRs). In 2017, the FDA approved pembrolizumab for use in patients with advanced or metastatic solid tumors with MSI-H or dMMR. However, MSI in HCC seems to be a rare event [64]. Therefore, the search for markers that predict the response of HCC to immunotherapy still needs further exploration.

PD-L1 expression is the first predictive biomarker in cancer immunotherapy [65]. Sangro et al. observed complete or partial tumor responses in both PD-L1-positive and PD-L1-negative patients treated with nivolumab monotherapy. The median OS of patients with high and low expression of PD-L1 was 28.1 (95% CI: 18.2–N.A) vs. 16.6 months (95% CI: 14.2–20.2), respectively [66]. However, the expression of PD-L1 is controversial in predicting the response to immunotherapy in HCC. Shrestha et al*.* [33] reported that only 65 of 751 HCC patients expressed PD-L1. Therefore, whether PD-L1 expression can be used to predict the efficacy of ICI in patients with HCC still needs further research.

TILs, including B cells, natural killer cells, and T cells, are an important part of body antitumor immunity. Studies have shown that TILs (especially CD8+ T cells) can be used as a predictor of primary resected liver tumors and as an independent indicator of survival and recurrence of metastatic liver tumors [67]. Katz et al. [68] demonstrated that for metastatic liver tumors, high levels of Treg infiltration had a suppressive effect on immunity. β-catenin is a multifunctional protein encoded by the *CTNNB1* gene, and mutations in β-catenin induced activation of WNT signaling and were associated with poor immune cell infiltration [69]. It has been suggested that there are two modes of lymphocyte infiltration in HCC. One type is noninfiltrating, characterized by tumors with mutations in *TP53* and *CTNNB1* genes, which are insensitive to immunotherapy, and are known as ‘cold tumors’. In contrast, lymphocyte-infiltrating tumors that do not have TP53 or CTNNB1 mutations and are also known as ‘hot tumors’ [70]. Harding et al. [71] analyzed the correlation between therapeutic response and the genome mutation spectrum of 27 HCC patients treated with ICIs and found that activating mutations in the WNT/β-catenin pathway were associated with lower DCR, shorter PFS, and shorter OS. Cellular diversity in tumors is a key factor in therapeutic failures and lethal outcomes of solid malignancies. Sangro et al. [66] found that the four inflammatory gene signature was associated with an improved ORR. Ma et al. [72] found that tumors with higher transcriptomic diversity were associated with worse OS.

Host-related markers and liquid biopsy biomarkers are also recent research hotspots. ESMO demonstrated the predictive value of the neutrophil-to-lymphocyte ratio (NLR) and platelet-to-lymphocyte ratio (PLR) in the treatment of liver cancer with nivolumab [73]. Zheng et al. reported that fecal samples from patients who responded to ICI (*n*=3) during anti-PD-1 immunotherapy for HCC showed a higher *Lactobacillus* content in their intestinal microbiota than those who did not (*n*=5). The intestinal microbiota was suggested for the first time to influence the efficacy of PD-1/PD-L1 in the treatment of HCC [74]. Imaging methods have also been used to evaluate the response of advanced HCC to immunotherapy. For example, Qayyum et al*.* [75] used magnetic resonance elastography (MRE) to evaluate the therapeutic effect of immunotherapy in advanced HCC. In this prospective study, they found that early changes in tumor stiffness in MRE may be an important factor in evaluating the efficacy of treatment for advanced HCC. Age was also a predictor of the ICI response. Studies have shown that the ORR of patients under 65 years of age for anti-PD-1 treatment is low [76]. The reason may be that elderly patients have a less active immune system and anti-PD-1/PD-L1 antibodies can restore lost antitumor immunity, so elderly patients may benefit more. With the deepening of research, an increasing number of prognostic biomarkers related to ICIs have been proposed (Table 3) [33–35,46,63,64,66–68,77–81].

**Table 3:** Biomarkers for ICI response reported in HCC

Biomarker	Association with clinical outcome	References
TMB	Positive or negative	[33,46,63,77,78]
MSI	Positive or unknown	[64]
PD-L1 expression in tumor	Irrelevant or positive	[33–35,66,79]
Soluble PD-L1	Negative	[80]
CD8+ T cells	Positive	[67]
Treg cells	Negative	[68]
WNT/β-catenin pathway activation	Negative	[71]
Transcriptomic diversity	Negative	[72]
NLR, PLR	Negative	[73]
*Lactobacillus*	Positive	[74]
Male sex	Positive	[76]
Age (>60 or >65 years)	Positive	[76,81]

## Immunotherapy and hyperprogressive disease

Cancer progression has been reported to be accelerated by an unexpected increase in the progression rate and tumor volume during immunotherapy, often leading to a significant reduction in survival time. This condition is therefore termed hyperprogressive disease (HPD). A more comprehensive definition is as follows: in immunotherapy, the time for tumor progression is less than 2 months, the tumor burden increases by more than 50% compared with the baseline period, and the tumor growth rate (TGR) after immunotherapy increases more than two-fold. The tumor flare caused by treatment is a paradoxical phenomenon and is a significant challenge for the management of immunotherapy in clinics. The reported incidence of HPD is between 4 and 29%, which may depend on the enrolled population and the type of tumor [82–84]. Although HPD frequently occurs in the context of ICB treatment, the mechanism of its occurrence has not been reasonably explained [85]. It is necessary to improve the understanding of the nature of this phenomenon in the clinic to accurately identify suitable patients for immunotherapy. Many studies have been conducted to identify clinical or molecular factors that can be used to predict HPD, such as hemoglobin, Child-Pugh Score, portal vein tumor thrombus (PVTT), NLR, MDM2, BIRC5, circulating tumor DNA (ctDNA), MMR etc. (Table 4) [86–99], although these factors require further validation in HCC and other cancers.

**Table 4:** Biomarkers for HPD after ICI therapy

Biomarker	Prognostic significance	References
Hemoglobin Child-Pugh score PVTT	Hemoglobin level, portal vein tumor thrombus (PVVT), and Child-Pugh score were significantly related to HPD	[86]
NLR	The high NLR was significantly associated with HPD, as the NLR value increased, the risk of HPD increased gradually in HCC	[87,88]
MDM2 BIRC5	MDM2 cooperated with BIRC5 to promote the HPD phenomenon in patients with advanced HCC	[89,100]
ctDNA	A high concentration of ctDNA was associated with a higher risk of HPD and poor PFS in NSCLC	[90]
Chemoattractant protein 1	Low serum monocyte chemoattractant protein was associated with HPD	[91]
EGFR	Overexpression of EGFR lowered the response rates to ICI therapy	[92]
BRCA2	Enriched mutations in the DNA repair gene *BRCA2* improved anti-PD-1 response in cancer	[93]
MMR	Deficiency of MMR predicted better prognosis in cancer	[94,95]
Treg	Activation of Treg promoted hyperprogression of cancer	[96]
T cells	Increased TPEX cell frequencies were associated with increased patient survival	[97]
MDSCs	Low frequency of MDSCs suggested that patients were more likely to respond to ipilimumab treatment	[98]
IFN-γ	IFN-γ-mediated inhibition of lung cancer by up-regulating the expression of PD-L1, leading to a favorable prognosis	[99]

Abbreviation: CRP, C-reactive protein.

## Conclusions and perspectives

Current studies have demonstrated that ICIs represented by anti-PD-1/PD-L1 and anti-CTLA-4 antibodies have shown good results in the clinical treatment of advanced HCC. ICI combination therapy with LRT or molecularly targeted drugs, such as TKIs, can further improve anticancer efficiency. How to effectively utilize the synergistic effects of different antitumor mechanisms will be the focus of future research and is expected to change the status of HCC treatment. In general, ICIs are safe and cause fewer AEs, such as skin reactions, immune diarrhea, liver and kidney toxicity, immune-related pneumonia, and gastrointestinal disorders. At the same time, the treatment process should be closely monitored, timely detection and treatment of adverse reactions should be performed, and unnecessary treatment interruptions should be reduced. Although ICIs have broad prospects for the treatment of HCC, their ORR is still relatively low. The discovery and application of biomarkers for the effect of ICB therapy will help clinicians effectively screen patients who would benefit from ICI treatment and make individualized treatment more precise. However, at present, biomarkers for ICI beneficiaries of liver cancer are still in the exploratory stage or lack strong evidence, and the combination of multiple biomarkers may be a new development trend. In the future, there is a need to develop more immunosuppressive agents, explore new therapies, and discover new prognostic biomarkers to achieve better treatment results. More RCTs with larger sample sizes are required to further validate the therapeutic results of ICIs for advanced HCC.

## Abbreviations

Ab: antibody
ADR: adverse reaction
AE: adverse event
APC: antigen-presenting cell
CTLA-4: cytotoxic T lymphocyte-associated antigen 4
DC: dendritic cell
DCR: disease control rate
dMMR: mismatch repair defect
ESMO: European Society of Medical Oncology
FDA: Food and Drug Administration
HBsAg: hepatitis B surface antigen
HCC: hepatocellular carcinoma
HCV: hepatitis C virus
HPD: hyperprogressive disease
ICB: immune checkpoint blockade
ICI: immune checkpoint inhibitor
ICP: immune checkpoint
LAG-3: lymphocyte activation gene-3
LRT: local regional therapy
MDSC: myeloid-derived suppressor cell
mOS: median overall survival
mPFS: median progression-free survival
MRE: magnetic resonance elastography
MSI: microsatellite instability
NLR: neutrophil-to-lymphocyte ratio
NSCLC: non-small-cell lung cancer
ORR: objective response rate
OS: overall survival
PD-1: programmed cell death 1
PD-L1: programmed death ligand 1
PFS: progression-free survival
RCT: randomized controlled trial
RFA: radiofrequency ablation
TACE: transcatheter arterial chemoembolization
TIGIT: T-cell immunoglobulin and ITIM domain protein
TIL: tumor-infiltrating lymphocyte
TIM-3: T-cell immunoglobulin mucin-3
TKI: tyrosine kinase inhibitor
TMB: tumor mutation burden
TME: tumor microenvironment
Treg: regulatory T cell

## References

[1] Sung H., Ferlay J. and Siegel R.L. (2021) Global Cancer Statistics 2020: GLOBOCAN estimates of incidence and mortality worldwide for 36 cancers in 185 countries. CA Cancer J. Clin. 71, 209–249 10.3322/caac.21660 33538338

[2] Singal A.G., Lampertico P. and Nahon P. (2020) Epidemiology and surveillance for hepatocellular carcinoma: new trends. J. Hepatol. 72, 250–261 10.1016/j.jhep.2019.08.025 31954490 PMC6986771

[3] Akoad M.E. and Pomfret E.A. (2015) Surgical resection and liver transplantation for hepatocellular carcinoma. Clin. Liver Dis. 19, 381–399 10.1016/j.cld.2015.01.007 25921669

[4] Brunetti O., Gnoni A., Licchetta A., Longo V., Calabrese A., Argentiero A. et al. (2019) Predictive and prognostic factors in HCC patients treated with sorafenib. Medicina (B. Aires) 55, 707 10.3390/medicina55100707 31640191 PMC6843290

[5] Raoul J.L., Forner A., Bolondi L., Cheung T.T., Kloeckner R. and de Baere T. (2019) Updated use of TACE for hepatocellular carcinoma treatment: how and when to use it based on clinical evidence. Cancer Treat. Rev. 72, 28–36 10.1016/j.ctrv.2018.11.002 30447470

[6] Li W. and Ni C.F. (2019) Current status of the combination therapy of transarterial chemoembolization and local ablation for hepatocellular carcinoma. Abdom. Radiol. (N.Y.) 44, 2268–2275 10.1007/s00261-019-01943-2 31016345

[7] Meyer T. (2020) Stereotactic body radiotherapy for hepatocellular carcinoma - still searching for a role. J. Hepatol. 73, 15–16 10.1016/j.jhep.2020.04.019 32386956

[8] Burki T.K. (2019) Hepatic arterial chemotherapy for hepatocellular carcinoma. Lancet Oncol. 20, e301 10.1016/S1470-2045(19)30343-2 31104912

[9] Qi X., Lam S.S., Liu D., Kim D.Y., Ma L., Alleruzzo L. et al. (2016) Development of inCVAX, in situ cancer vaccine, and its immune response in mice with hepatocellular cancer. J. Clin. Cell. Immunol. 7, 438 10.4172/2155-9899.1000438 27656328 PMC5027967

[10] Cai X.R., Li X., Lin J.X., Wang T.T., Dong M., Chen Z.H. et al. (2017) Autologous transplantation of cytokine-induced killer cells as an adjuvant therapy for hepatocellular carcinoma in Asia: an update meta-analysis and systematic review. Oncotarget 8, 31318–31328 10.18632/oncotarget.15454 28412743 PMC5458210

[11] Liu Y. and Cao X. (2016) Immunosuppressive cells in tumor immune escape and metastasis. J. Mol. Med. (Berl.) 94, 509–522 10.1007/s00109-015-1376-x 26689709

[12] Beatty G.L. and Gladney W.L. (2015) Immune escape mechanisms as a guide for cancer immunotherapy. Clin. Cancer Res. 21, 687–692 10.1158/1078-0432.CCR-14-1860 25501578 PMC4334715

[13] Ikeda M., Morizane C., Ueno M., Okusaka T., Ishii H. and Furuse J. (2018) Chemotherapy for hepatocellular carcinoma: current status and future perspectives. Jpn. J. Clin. Oncol. 48, 103–114 10.1093/jjco/hyx180 29253194

[14] Kuol N., Stojanovska L., Nurgali K. and Apostolopoulos V. (2018) PD-1/PD-L1 in disease. Immunotherapy 10, 149–160 10.2217/imt-2017-0120 29260623

[15] Puza C.J., Bressler E.S., Terando A.M., Howard J.H., Brown M.C., Hanks B. et al. (2019) The emerging role of surgery for patients with advanced melanoma treated with immunotherapy. J. Surg. Res. 236, 209–215 10.1016/j.jss.2018.11.045 30694757

[16] Ribas A. and Wolchok J.D. (2018) Cancer immunotherapy using checkpoint blockade. Science 359, 1350–1355 10.1126/science.aar4060 29567705 PMC7391259

[17] Maruhashi T. and Sugiura D. (2020) LAG-3: from molecular functions to clinical applications. *J. Immunother. Cancer* 8, e001014 10.1136/jitc-2020-001014 32929051 PMC7488795

[18] Sabatos C.A., Chakravarti S., Cha E., Schubart A., Sánchez-Fueyo A., Zheng X.X. et al. (2003) Interaction of Tim-3 and Tim-3 ligand regulates T helper type 1 responses and induction of peripheral tolerance. Nat. Immunol. 4, 1102–1110 10.1038/ni988 14556006

[19] Koyama S., Akbay E.A., Li Y.Y., Herter-Sprie G.S., Buczkowski K.A., Richards W.G. et al. (2016) Adaptive resistance to therapeutic PD-1 blockade is associated with upregulation of alternative immune checkpoints. Nat. Commun. 7, 10501 10.1038/ncomms10501 26883990 PMC4757784

[20] Anderson A.C., Joller N. and Kuchroo V.K. (2016) Lag-3, Tim-3, and TIGIT: co-inhibitory receptors with specialized functions in immune regulation. Immunity 44, 989–1004 10.1016/j.immuni.2016.05.001 27192565 PMC4942846

[21] Cheng H., Sun G., Chen H., Li Y., Han Z., Li Y. et al. (2019) Trends in the treatment of advanced hepatocellular carcinoma: immune checkpoint blockade immunotherapy and related combination therapies. Am. J. Cancer Res. 9, 1536–1545 31497341 PMC6726979

[22] Doherty D.G. (2016) Immunity, tolerance and autoimmunity in the liver: a comprehensive review. J. Autoimmun. 66, 60–75 10.1016/j.jaut.2015.08.020 26358406

[23] Najafi M., Farhood B. and Mortezaee K. (2019) Contribution of regulatory T cells to cancer: a review. *J. Cell Physiol.* 234, 7983–7993 10.1002/jcp.27553 30317612

[24] Valzasina B., Piconese S., Guiducci C. and Colombo M.P. (2006) Tumor-induced expansion of regulatory T cells by conversion of CD4+CD25- lymphocytes is thymus and proliferation independent. Cancer Res. 66, 4488–4495 10.1158/0008-5472.CAN-05-4217 16618776

[25] Xu M., Zhao Z., Song J., Lan X., Lu S., Chen M. et al. (2017) Interactions between interleukin-6 and myeloid-derived suppressor cells drive the chemoresistant phenotype of hepatocellular cancer. Exp. Cell Res. 351, 142–149 10.1016/j.yexcr.2017.01.008 28109867

[26] Lu L.C., Chang C.J. and Hsu C.H. (2019) Targeting myeloid-derived suppressor cells in the treatment of hepatocellular carcinoma: current state and future perspectives. J. Hepatocell. Carcinoma 6, 71–84 10.2147/JHC.S159693 31123667 PMC6511249

[27] Greten T.F. and Sangro B. (2017) Targets for immunotherapy of liver cancer. J. Hepatol. S0168–8278, 32287–0 10.1016/j.jhep.2017.09.007 28923358 PMC5857416

[28] Huang M., He M., Guo Y., Li H., Shen S., Xie Y. et al. (2020) The influence of immune heterogeneity on the effectiveness of immune checkpoint inhibitors in multifocal hepatocellular carcinomas. Clin. Cancer Res. 26, 4947–4957 10.1158/1078-0432.CCR-19-3840 32527942

[29] Zhou G., Sprengers D., Boor P.P.C., Doukas M., Schutz H., Mancham S. et al. (2017) Antibodies against immune checkpoint molecules restore functions of tumor-infiltrating T cells in hepatocellular carcinomas. Gastroenterology 153, 1107–1119 e10 10.1053/j.gastro.2017.06.017 28648905

[30] Rowshanravan B. and Halliday N. (2018) CTLA-4: a moving target in immunotherapy. Blood 131, 58–67 10.1182/blood-2017-06-741033 29118008 PMC6317697

[31] Siu L.L., Ivy S.P., Dixon E.L., Gravell A.E., Reeves S.A. and Rosner G.L. (2017) Challenges and opportunities in adapting clinical trial design for immunotherapies. Clin. Cancer Res. 23, 4950–4958 10.1158/1078-0432.CCR-16-3079 28864723 PMC5669041

[32] Baumeister S.H., Freeman G.J., Dranoff G. and Sharpe A.H. (2016) Coinhibitory pathways in immunotherapy for cancer. Annu. Rev. Immunol. 34, 539–573 10.1146/annurev-immunol-032414-112049 26927206

[33] Shrestha R., Prithviraj P., Anaka M., Bridle K.R., Crawford D.H.G., Dhungel B. et al. (2018) Monitoring immune checkpoint regulators as predictive biomarkers in hepatocellular carcinoma. Front. Oncol. 8, 269 10.3389/fonc.2018.00269 30057891 PMC6053505

[34] El-Khoueiry A.B., Sangro B., Yau T., Crocenzi T.S., Kudo M., Hsu C. et al. (2017) Nivolumab in patients with advanced hepatocellular carcinoma (CheckMate 040): an open-label, non-comparative, phase 1/2 dose escalation and expansion trial. Lancet North Am. Ed. 389, 2492–2502 10.1016/S0140-6736(17)31046-2 PMC7539326 28434648

[35] Zhu A.X., Finn R.S., Edeline J., Cattan S., Ogasawara S., Palmer D. et al. (2018) Pembrolizumab in patients with advanced hepatocellular carcinoma previously treated with sorafenib (KEYNOTE-224): a non-randomised, open-label phase 2 trial. Lancet Oncol. 19, 940–952 10.1016/S1470-2045(18)30351-6 29875066

[36] Finn R.S., Ryoo B.Y., Merle P., Kudo M., Bouattour M., Lim H.Y. et al. (2020) Pembrolizumab as second-line therapy in patients with advanced hepatocellular carcinoma in KEYNOTE-240: a randomized, double-blind, phase III trial. J. Clin. Oncol. 38, 193–202 10.1200/JCO.19.01307 31790344

[37] Finn R.S., Qin S., Ikeda M., Galle P.R., Ducreux M., Kim T.Y. et al. (2020) Atezolizumab plus bevacizumab in unresectable hepatocellular carcinoma. N. Engl. J. Med. 382, 1894–1905 10.1056/NEJMoa1915745 32402160

[38] Finkelmeier F., Czauderna C., Perkhofer L., Ettrich T.J., Trojan J., Weinmann A. et al. (2019) Feasibility and safety of nivolumab in advanced hepatocellular carcinoma: real-life experience from three German centers. J. Cancer Res. Clin. Oncol. 145, 253–259 10.1007/s00432-018-2780-8 30374657 PMC11810178

[39] Qin S., Ren Z., Meng Z., Chen Z., Chai X., Xiong J. et al. (2020) Camrelizumab in patients with previously treated advanced hepatocellular carcinoma: a multicentre, open-label, parallel-group, randomised, phase 2 trial. Lancet Oncol. 21, 571–580 10.1016/S1470-2045(20)30011-5 32112738

[40] Segal N.H., Ou S.I., Balmanoukian A., Fury M.G., Massarelli E., Brahmer J.R. et al. (2019) Safety and efficacy of durvalumab in patients with head and neck squamous cell carcinoma: results from a phase I/II expansion cohort. Eur. J. Cancer 109, 154–161 10.1016/j.ejca.2018.12.029 30731276

[41] Barlesi F., Vansteenkiste J., Spigel D., Ishii H., Garassino M., de Marinis F. et al. (2018) Avelumab versus docetaxel in patients with platinum-treated advanced non-small-cell lung cancer (JAVELIN Lung 200): an open-label, randomised, phase 3 study. Lancet Oncol. 19, 1468–1479 10.1016/S1470-2045(18)30673-9 30262187

[42] Sangro B., Gomez-Martin C., de la Mata M., Inarrairaegui M., Garralda E., Barrera P. et al. (2013) A clinical trial of CTLA-4 blockade with tremelimumab in patients with hepatocellular carcinoma and chronic hepatitis C. J. Hepatol. 59, 81–88 10.1016/j.jhep.2013.02.022 23466307

[43] Yau T., Kang Y.K., Kim T.Y., El-Khoueiry A.B., Santoro A., Sangro B. et al. (2020) Efficacy and safety of nivolumab plus ipilimumab in patients with advanced hepatocellular carcinoma previously treated with sorafenib: The CheckMate 040 randomized clinical trial. JAMA Oncol. 6, e204564 10.1001/jamaoncol.2020.4564 33001135 PMC7530824

[44] Feun L.G., Li Y.Y., Wu C., Wangpaichitr M., Jones P.D., Richman S.P. et al. (2019) Phase 2 study of pembrolizumab and circulating biomarkers to predict anticancer response in advanced, unresectable hepatocellular carcinoma. Cancer 125, 3603–3614 10.1002/cncr.32339 31251403 PMC7592647

[45] Kelley R.K., Sangro B., Harris W. and Ikeda M. (2021) Safety, efficacy, and pharmacodynamics of tremelimumab plus durvalumab for patients with unresectable hepatocellular carcinoma: randomized expansion of a Phase I/II study. 39, 2991–3001 10.1200/JCO.20.03555 34292792 PMC8445563

[46] Lee M.S., Ryoo B.Y., Hsu C.H., Numata K., Stein S., Verret W. et al. (2020) Atezolizumab with or without bevacizumab in unresectable hepatocellular carcinoma (GO30140): an open-label, multicentre, phase 1b study. Lancet Oncol. 21, 808–820 10.1016/S1470-2045(20)30156-X 32502443

[47] Emens L.A. (2012) Breast cancer immunobiology driving immunotherapy: vaccines and immune checkpoint blockade. Expert Rev. Anticancer Ther. 12, 1597–1611 10.1586/era.12.147 23253225 PMC3587160

[48] Wright K. (2020) FDA approves nivolumab plus ipilimumab for the treatment of advanced HCC. Oncology (Huntingt.) 34, 693606 32293694

[49] Kudo M. (2017) Immuno-oncology in hepatocellular carcinoma: 2017 update. Oncology 93, 147–159 10.1159/000481245 29258079

[50] Chiang C.L., Chan A.C.Y., Chiu K.W.H. and Kong F.S. (2019) Combined Stereotactic body radiotherapy and checkpoint inhibition in unresectable hepatocellular carcinoma: a potential synergistic treatment strategy. Front. Oncol. 9, 1157 10.3389/fonc.2019.01157 31799176 PMC6874138

[51] Yu J.I., Lee S.J., Lee J., Lim H.Y., Paik S.W., Yoo G.S. et al. (2019) Clinical significance of radiotherapy before and/or during nivolumab treatment in hepatocellular carcinoma. Cancer Med. 8, 6986–6994 10.1002/cam4.2570 31588679 PMC6853810

[52] Bruix J., Sala M. and Llovet J.M. (2004) Chemoembolization for hepatocellular carcinoma. Gastroenterology 127, S179–S188 10.1053/j.gastro.2004.09.032 15508083

[53] Singh P. and Toom S. (2020) The immune modulation effect of locoregional therapies and its potential synergy with immunotherapy in hepatocellular carcinoma. *J. Hepatocell. Carcinoma* 7, 11–17 10.2147/JHC.S187121 32104669 PMC7022138

[54] Zerbini A., Pilli M., Laccabue D., Pelosi G., Molinari A., Negri E. et al. (2010) Radiofrequency thermal ablation for hepatocellular carcinoma stimulates autologous NK-cell response. Gastroenterology 138, 1931–1942 10.1053/j.gastro.2009.12.051 20060829

[55] Duffy A.G., Ulahannan S.V., Makorova-Rusher O., Rahma O., Wedemeyer H., Pratt D. et al. (2017) Tremelimumab in combination with ablation in patients with advanced hepatocellular carcinoma. J. Hepatol. 66, 545–551 10.1016/j.jhep.2016.10.029 27816492 PMC5316490

[56] Cui H., Dai G. and Guan J. (2020) Programmed cell death protein-1 (PD-1)-targeted immunotherapy for advanced hepatocellular carcinoma in real world. Onco Targets Ther. 13, 143–149 10.2147/OTT.S234868 32021262 PMC6955600

[57] Scheiner B., Kirstein M.M., Hucke F., Finkelmeier F., Schulze K., von Felden J. et al. (2019) Programmed cell death protein-1 (PD-1)-targeted immunotherapy in advanced hepatocellular carcinoma: efficacy and safety data from an international multicentre real-world cohort. Aliment. Pharmacol. Ther. 49, 1323–1333 10.1111/apt.15245 30980420 PMC6593858

[58] Chen K., Wei W., Liu L., Deng Z.J., Li L., Liang X.M. et al. (2021) Lenvatinib with or without immune checkpoint inhibitors for patients with unresectable hepatocellular carcinoma in real-world clinical practice. *Cancer Immunol. Immunother.* 10.1007/s00262-021-03060-w 34559308 PMC10992447

[59] Sung P.S., Jang J.W., Lee J., Lee S.K., Lee H.L., Yang H. et al. (2020) Real-world outcomes of nivolumab in patients with unresectable hepatocellular carcinoma in an endemic area of Hepatitis B virus infection. Front. Oncol. 10, 1043 10.3389/fonc.2020.01043 32695681 PMC7338665

[60] Liu B.J. and Gao S. (2021) Real-world study of hepatic artery infusion chemotherapy combined with anti-PD-1 immunotherapy and tyrosine kinase inhibitors for advanced hepatocellular carcinoma. *Immunotherapy* 13, 1395–1405 10.2217/imt-2021-0192 34607482

[61] Xie D., Sun Q., Wang X., Zhou J., Fan J., Ren Z. et al. (2021) Immune checkpoint inhibitor plus tyrosine kinase inhibitor for unresectable hepatocellular carcinoma in the real world. Ann. Transl. Med. 9, 652 10.21037/atm-20-7037 33987350 PMC8106062

[62] Goodman A.M., Kato S., Bazhenova L., Patel S.P., Frampton G.M., Miller V. et al. (2017) Tumor mutational burden as an independent predictor of response to immunotherapy in diverse cancers. Mol. Cancer Ther. 16, 2598–2608 10.1158/1535-7163.MCT-17-0386 28835386 PMC5670009

[63] Ang C., Klempner S.J., Ali S.M., Madison R., Ross J.S., Severson E.A. et al. (2019) Prevalence of established and emerging biomarkers of immune checkpoint inhibitor response in advanced hepatocellular carcinoma. Oncotarget 10, 4018–4025 10.18632/oncotarget.26998 31258846 PMC6592287

[64] Goumard C., Desbois-Mouthon C., Wendum D., Calmel C., Merabtene F., Scatton O. et al. (2017) Low levels of microsatellite instability at simple repeated sequences commonly occur in human hepatocellular carcinoma. Cancer Genomics Proteomics 14, 329–339 28871000 10.21873/cgp.20043 PMC5611519

[65] Patel S.P. and Kurzrock R. (2015) PD-L1 expression as a predictive biomarker in cancer immunotherapy. Mol. Cancer Ther. 14, 847–856 10.1158/1535-7163.MCT-14-0983 25695955

[66] Sangro B., Melero I., Wadhawan S., Finn R.S., Abou-Alfa G.K., Cheng A.L. et al. (2020) Association of inflammatory biomarkers with clinical outcomes in nivolumab-treated patients with advanced hepatocellular carcinoma. J. Hepatol. 73, 1460–1469 10.1016/j.jhep.2020.07.026 32710922 PMC7751218

[67] Khan H., Pillarisetty V.G. and Katz S.C. (2014) The prognostic value of liver tumor T cell infiltrates. J. Surg. Res. 191, 189–195 10.1016/j.jss.2014.06.001 25033707 PMC4134707

[68] Katz S.C., Bamboat Z.M., Maker A.V., Shia J., Pillarisetty V.G., Yopp A.C. et al. (2013) Regulatory T cell infiltration predicts outcome following resection of colorectal cancer liver metastases. Ann. Surg. Oncol. 20, 946–955 10.1245/s10434-012-2668-9 23010736 PMC3740360

[69] Berraondo P. and Ochoa M.C. (2019) Immune desertic landscapes in hepatocellular carcinoma shaped by β-Catenin activation. *Cancer Discov.* 9, 1003–1005 10.1158/2159-8290.CD-19-0696 31371324

[70] Sia D., Jiao Y., Martinez-Quetglas I., Kuchuk O., Villacorta-Martin C., Castro de Moura M. et al. (2017) Identification of an immune-specific class of hepatocellular carcinoma, based on molecular features. Gastroenterology 153, 812–826 10.1053/j.gastro.2017.06.007 28624577 PMC12166766

[71] Harding J.J., Nandakumar S., Armenia J., Khalil D.N., Albano M., Ly M. et al. (2019) Prospective genotyping of hepatocellular carcinoma: clinical implications of next-generation sequencing for matching patients to targeted and immune therapies. *Clin. Cancer Res.* 25, 2116–2126 10.1158/1078-0432.CCR-18-2293 30373752 PMC6689131

[72] Ma L., Hernandez M.O., Zhao Y., Mehta M., Tran B., Kelly M. et al. (2019) Tumor cell biodiversity drives microenvironmental reprogramming in liver cancer. Cancer Cell 36, 418.e6–430.e6 10.1016/j.ccell.2019.08.007 31588021 PMC6801104

[73] Dharmapuri S., Özbek U. and Lin J.Y. (2020) Predictive value of neutrophil to lymphocyte ratio and platelet to lymphocyte ratio in advanced hepatocellular carcinoma patients treated with anti-PD-1 therapy. Cancer Med. 9, 4962–4970 10.1002/cam4.3135 32419290 PMC7367631

[74] Zheng Y., Wang T., Tu X., Huang Y., Zhang H., Tan D. et al. (2019) Gut microbiome affects the response to anti-PD-1 immunotherapy in patients with hepatocellular carcinoma. J. Immunother. Cancer 7, 193 10.1186/s40425-019-0650-9 31337439 PMC6651993

[75] Qayyum A., Hwang K.P., Stafford J., Verma A., Maru D.M., Sandesh S. et al. (2019) Immunotherapy response evaluation with magnetic resonance elastography (MRE) in advanced HCC. J. Immunother. Cancer 7, 329 10.1186/s40425-019-0766-y 31779702 PMC6883599

[76] Nosrati A., Tsai K.K., Goldinger S.M., Tumeh P., Grimes B., Loo K. et al. (2017) Evaluation of clinicopathological factors in PD-1 response: derivation and validation of a prediction scale for response to PD-1 monotherapy. Br. J. Cancer 116, 1141–1147 10.1038/bjc.2017.70 28324889 PMC5418446

[77] Xu J., Zhang Y., Jia R., Yue C., Chang L., Liu R. et al. (2019) Anti-PD-1 antibody SHR-1210 combined with apatinib for advanced hepatocellular carcinoma, gastric, or esophagogastric junction cancer: an open-label, dose escalation and expansion study. Clin. Cancer Res. 25, 515–523 30348638 10.1158/1078-0432.CCR-18-2484

[78] Cai H., Zhang Y., Zhang H., Cui C., Li C. and Lu S. (2020) Prognostic role of tumor mutation burden in hepatocellular carcinoma after radical hepatectomy. J. Surg. Oncol. 121, 1007–1014 10.1002/jso.25859 31995247

[79] Chen C.L., Pan Q.Z., Zhao J.J., Wang Y., Li Y.Q., Wang Q.J. et al. (2016) PD-L1 expression as a predictive biomarker for cytokine-induced killer cell immunotherapy in patients with hepatocellular carcinoma. Oncoimmunology 5, e1176653 10.1080/2162402X.2016.1176653 27622026 PMC5006896

[80] Zhou J., Mahoney K.M., Giobbie-Hurder A., Zhao F., Lee S., Liao X. et al. (2017) Soluble PD-L1 as a biomarker in malignant melanoma treated with checkpoint blockade. Cancer Immunol. Res. 5, 480–492 10.1158/2326-6066.CIR-16-0329 28522460 PMC5642913

[81] Kugel C.H. III, Douglass S.M., Webster M.R., Kaur A., Liu Q., Yin X. et al. (2018) Age correlates with response to anti-PD1, reflecting age-related differences in intratumoral effector and regulatory T-cell populations. Clin. Cancer Res. 24, 5347–5356 10.1158/1078-0432.CCR-18-1116 29898988 PMC6324578

[82] Lo Russo G., Moro M., Sommariva M., Cancila V., Boeri M., Centonze G. et al. (2019) Antibody-Fc/FcR interaction on macrophages as a mechanism for hyperprogressive disease in non-small cell lung cancer subsequent to PD-1/PD-L1 blockade. Clin. Cancer Res. 25, 989–999 10.1158/1078-0432.CCR-18-1390 30206165

[83] Ferrara R., Mezquita L., Texier M., Lahmar J., Audigier-Valette C., Tessonnier L. et al. (2018) Hyperprogressive disease in patients with advanced non-small cell lung cancer treated with PD-1/PD-L1 inhibitors or with single-agent chemotherapy. JAMA Oncol. 4, 1543–1552 10.1001/jamaoncol.2018.3676 30193240 PMC6248085

[84] Champiat S., Dercle L., Ammari S., Massard C., Hollebecque A., Postel-Vinay S. et al. (2017) Hyperprogressive disease is a new pattern of progression in cancer patients treated by anti-PD-1/PD-L1. Clin. Cancer Res. 23, 1920–1928 10.1158/1078-0432.CCR-16-1741 27827313

[85] Champiat S., Ferrara R., Massard C., Besse B., Marabelle A., Soria J.C. et al. (2018) Hyperprogressive disease: recognizing a novel pattern to improve patient management. Nat. Rev. Clin. Oncol. 15, 748–762 10.1038/s41571-018-0111-2 30361681

[86] Zhang L., Wu L., Chen Q., Zhang B., Liu J., Liu S. et al. (2021) Predicting hyperprogressive disease in patients with advanced hepatocellular carcinoma treated with anti-programmed cell death 1 therapy. eClin. Med. 31, 100673 10.1016/j.eclinm.2020.100673 33554079 PMC7846667

[87] Choi W.M., Kim J.Y., Choi J., Lee D., Shim J.H., Lim Y.S. et al. (2021) Kinetics of the neutrophil-lymphocyte ratio during PD-1 inhibition as a prognostic factor in advanced hepatocellular carcinoma. Liver Int. 41, 2189–2199 10.1111/liv.14932 33966338

[88] Kim C.G., Kim C., Yoon S.E., Kim K.H., Choi S.J., Kang B. et al. (2021) Hyperprogressive disease during PD-1 blockade in patients with advanced hepatocellular carcinoma. J. Hepatol. 74, 350–359 10.1016/j.jhep.2020.08.010 32810553

[89] Wu L., Quan W., Luo Q., Pan Y., Peng D. and Zhang G. (2020) Identification of an immune-related prognostic predictor in hepatocellular carcinoma. Front. Mol. Biosci. 7, 567950 10.3389/fmolb.2020.567950 33195412 PMC7542239

[90] Chen Y., Li X., Liu G., Chen S., Xu M., Song L. et al. (2020) ctDNA concentration, MIKI67 mutations and hyper-progressive disease related gene mutations are prognostic markers for camrelizumab and apatinib combined multiline treatment in advanced NSCLC. Front. Oncol. 10, 1706 10.3389/fonc.2020.01706 33014846 PMC7509428

[91] Lu Z., Zou J., Hu Y., Li S., Zhou T., Gong J. et al. (2019) Serological markers associated with response to immune checkpoint blockade in metastatic gastrointestinal tract cancer. JAMA Netw. Open 2, e197621 10.1001/jamanetworkopen.2019.7621 31339548 PMC6659353

[92] Gainor J.F., Shaw A.T., Sequist L.V., Fu X., Azzoli C.G., Piotrowska Z. et al. (2016) EGFR mutations and ALK rearrangements are associated with low response rates to PD-1 pathway blockade in non-small cell lung cancer: a retrospective analysis. Clin. Cancer Res. 22, 4585–4593 10.1158/1078-0432.CCR-15-3101 27225694 PMC5026567

[93] Hugo W., Zaretsky J.M., Sun L., Song C., Moreno B.H., Hu-Lieskovan S. et al. (2016) Genomic and transcriptomic features of response to anti-PD-1 therapy in metastatic melanoma. Cell 165, 35–44 10.1016/j.cell.2016.02.065 26997480 PMC4808437

[94] Lee V. and Le D.T. (2016) Efficacy of PD-1 blockade in tumors with MMR deficiency. Immunotherapy 8, 1–3 10.2217/imt.15.97 26643016

[95] Zhao P., Li L., Jiang X. and Li Q. (2019) Mismatch repair deficiency/microsatellite instability-high as a predictor for anti-PD-1/PD-L1 immunotherapy efficacy. J. Hematol. Oncol. 12, 54 10.1186/s13045-019-0738-1 31151482 PMC6544911

[96] Kamada T., Togashi Y., Tay C., Ha D., Sasaki A., Nakamura Y. et al. (2019) PD-1(+) regulatory T cells amplified by PD-1 blockade promote hyperprogression of cancer. Proc. Natl. Acad. Sci. U.S.A. 116, 9999–10008 10.1073/pnas.1822001116 31028147 PMC6525547

[97] Kallies A., Zehn D. and Utzschneider D.T. (2020) Precursor exhausted T cells: key to successful immunotherapy? Nat. Rev. Immunol. 20, 128–136 10.1038/s41577-019-0223-7 31591533

[98] Meyer C., Cagnon L., Costa-Nunes C.M., Baumgaertner P., Montandon N., Leyvraz L. et al. (2014) Frequencies of circulating MDSC correlate with clinical outcome of melanoma patients treated with ipilimumab. Cancer Immunol. Immunother. 63, 247–257 10.1007/s00262-013-1508-5 24357148 PMC11029062

[99] Gao Y., Yang J., Cai Y., Fu S., Zhang N., Fu X. et al. (2018) IFN-gamma-mediated inhibition of lung cancer correlates with PD-L1 expression and is regulated by PI3K-AKT signaling. Int. J. Cancer 143, 931–943 10.1002/ijc.31357 29516506

[100] Kato S., Goodman A., Walavalkar V., Barkauskas D.A., Sharabi A. and Kurzrock R. (2017) Hyperprogressors after immunotherapy: analysis of genomic alterations associated with accelerated growth rate. Clin. Cancer Res. 23, 4242–4250 10.1158/1078-0432.CCR-16-3133 28351930 PMC5647162

